# The Pharmacogenomic HLA Biomarker Associated to Adverse Abacavir Reactions: Comparative Analysis of Different Genotyping Methods

**DOI:** 10.2174/138920212800793311

**Published:** 2012-06

**Authors:** Laura Stocchi, Raffaella Cascella, Stefania Zampatti, Antonella Pirazzoli, Giuseppe Novelli, Emiliano Giardina

**Affiliations:** 1 Università degli Studi di Roma Tor Vergata, Centro di Eccellenza per lo Studio del Rischio Genomico in Patologie Complesse Multifattoriali, Roma, Italy; 2 ViiV Healthcare, Verona, Italy; 3 Agenzia di Valutazione del Sistema Universitario e della Ricerca (ANVUR); 4 Ospedale San Pietro, Fatebenefratelli, Roma, Italy

**Keywords:** HLA-B*57:01, abacavir, hypersensitivity reaction (HSR), pharmacogenomics.

## Abstract

Many pharmacogenomic biomarkers (PGBM) were identified and translated into clinical practice, affecting the usage of drugs *via *label updates. In this context, abacavir is one of the most brilliant examples of pharmacogenetic studies translated into clinical practice. Pharmacogenetic studies have revealed that abacavir HSRs are highly associated with the major histocompatibility complex class I. Large studies established the effectiveness of prospective HLA-B*57:01 screening to prevent HSRs to abacavir. Accordingly to these results the abacavir label has been modified: the European Medicines Agency (EMA) and the FDA recommend/suggested that the administration of abacavir must be preceded by a specific genotyping test. The *HLA* locus is extremely polymorphic, exhibiting many closely related alleles, making it difficult to discriminate HLA-B*57:01 from other related alleles, and a number of different molecular techniques have been developed recently to detect the presence of HLA-B*57:01. In this review, we provide a summary of the available techniques used by laboratories to genotype HLA-B*57:01, outlining the scientific and pharmacoeconomics pros and cons.

The increasing knowledge of variation within the human genome is being used for the development of personalized and stratified medicine, with the aim to decrease the number of adverse medicinal product reactions and to increase the efficacy of medicinal product therapy to optimize medicines benefit/risk ratio. Recent years have witnessed much progress in the field of pharmacogenetics, with an increasing number of publications and several regulatory changes that promoted the overall usefulness and significance of pharmacogenetics in clinical management and novel drug discovery and development [1]. Recently, many pharmacogenomic biomarker (PGBM) were identified and translated into clinical practice, affecting the usage of drugs such as carbamazepine, warfarin, and abacavir *via *drug label updates. In this context, abacavir is one of the most brilliant examples of pharmacogenetic studies translated into clinical practice [2]. Abacavir sulfate is a nucleoside reverse-transcriptase inhibitor that is indicated for the treatment of HIV infection. Abacavir is available as a single entity formulation (Ziagen^®^, ViiV Healthcare) or in combination with other retroviral agents such as lamivudine (Epzicom/Kivexa^®^, ViiV Healthcare) and zidovudine (Trizivir^®^, ViiV Healthcare). Abacavir has been approved for use by the US Food and Drug Administration (FDA) since 1998, and it received full marketing approval in Europe in 1999. It has been used by almost 1 million patients infected with HIV during the past decade [3], and it is generally well tolerated, although 5% of patients develop an allergic reaction, usually within the 6 weeks of starting abacavir. Such a hypersensitivity reaction (HSR) is a multiorgan syndrome characterized by two or more clinical signs including fever, rash, as well as gastrointestinal, respiratory and constitutional symptoms [4]. If a patient experiences an HSR, then abacavir is discontinued, and symptoms generally resolve within 72 h [5]. Restarting abacavir is contraindicated, as it can result in a potentially life-threatening reaction and even death [6,7]. 

Pharmacogenetic studies have revealed that abacavir HSRs are highly associated with the major histocompatibility complex class I, and in 2002, two independent groups demonstrated a strong association between the HLA-B*57:01 allele and abacavir HSR [8,9]. PREDICT-1 is a prospective, randomized, and double blind study that assessed the incidence of abacavir HSR in HIV-1-positive patients [10]. As a result, it has been estimated that 61% of HLA-B*57:01-positive patients developed HSR to abacavir. SHAPE [11] is a retrospective study based on case-control studies. The SHAPE study estimated the incidence of HSR to abacavir in black and white subjects in the United States. The results confirmed the strong association between HLA-B*57:01 and HSR in both analyzed groups of patients. Overall, a large number of studies demonstrated that abacavir HSR is associated with the major histocompatibility complex class 1 allele HLA-B*57:01 [8,12,13]. Large studies established the effectiveness of prospective HLA-B*57:01 screening to prevent HSRs to abacavir [8-11], and more recently, two large randomized studies demonstrated a 100% negative predictive value of HLA-B*57:01 screening in white and black populations [10-11]. Accordingly to these results the abacavir label has been modified: the European Medicines Agency (EMA) and the FDA recommend/suggested that the administration of abacavir must be preceded by a specific genotyping test. The *HLA* locus is extremely polymorphic, exhibiting many closely related alleles, making it difficult to discriminate HLA-B*57:01 from other related alleles, and a number of different molecular techniques have been developed recently to detect the presence of HLA-B*57:01. These strategies involve both serological and molecular methods, such as sequence-specific oligonucleotide probe hybridization (SSOP), DNA sequence-based typing (SBT), sequence-specific primers polymerase chain reaction (SSP-PCR), flow cytometry, allele-specific polymerase chain reaction (AS-PCR), quantitative PCR (Q-PCR), and SSP-PCR with fluorescence detection through capillary electrophoresis (CE). In this review, we provide a summary of the available techniques used by laboratories to genotype HLA-B*57:01, outlining the scientific and pharmacoeconomics pros and cons. 

## SEROLOGICAL APPROACHES 

The serological approaches are characterized by detection of antibodies in blood serum. There are several serological techniques (ELISA, agglutination, precipitation, complement-fixation, and fluorescent antibodies) that can be used depending on the antibodies being studied. Standard serological approaches used to detect HLA-B*57 lack specificity, as commercially available monoclonal antibodies cross-react with HLA-B*57 and HLA-B*58 subtypes [14]. Serological methods require intact cells and may also result in false-negative results if HLA-class I molecules are downregulated during infection. Nowadays serological tests have been replaced by flow cytometry for analysis and sorting of blood cells. 

## FLOW CYTOMETRY (FCM)

The flow cytometry (FCM) is a technique for counting and examining cells and chromosomes. Flow cytometry was the first approach to describe immune cell subsets [15]. Indeed a lot of the T-cell subsets, altered by HIV, were first identified by this method [16]. Flow cytometry is used to describe the first changes in blood cells and afterwards to confirm the loss of CD4+ T-cells [17]. This methodology can also be used to investigate the presence of HLA-B*57 locus. As flow cytometry requires blood samples it allows to analyze the same samples for both CD4+ count and HLA-B*57 test. In 2006, a new flow cytometry-based test to type HLA-B*57:01 was developed [18]. The authors developed a specific protocol based on the use of a B17 monoclonal antibody to detect cell surface antigens on CD45^+^ lymphocytes. Commercially available B17 monoclonal antibodies recognizing all subgroups of HLA-B*57 and HLA-B*58 antigens are used on peripheral blood samples. Results can be obtained within 12–24 h of blood collection. Test results are based on the fluorescence intensity of lymphocytes, and the results can be stratified into three categories: a) B17 unequivocal, > 80% of lymphocytes were positive; b) not excluded, 50–70% of lymphocytes were positive; c) negative, < 20% of lymphocytes were positive. This method is 100% sensitive for the detection of HLA-B*57:01-positive individuals is but not 100% specific, as it cross-reacts with many *HLA* alleles within the B17 serological group. Thus, further molecular typing is required to determine the exact genotype of positive samples. The assay can be accomplished within 2–3 h of sample receipt with a cost of approximately $30 USD per patient (for the first step). In 2011 it has been developed [19] a monoclonal antibody (mAb, 3E12) specific for HLA-B*57 and HLA-B*58 strongly associated with clinically important immune phenotypes. A direct immunofluorescence (IF) using this monoclonal antibody can be applied for staining the peripheral blood mononuclear cell (PBMC) for the flow cytometry. As this methodology cannot type *HLA-B* locus at allele level, it can be used only to exclude the negative subjects, with patients positive to be further analyzed with high-resolution typing to discriminate HLA-B*57:01 allele from the other related alleles [19].

## MOLECULAR METHODS

### Sequence Specific Oligonucleotide Probes (SSOP)

The Sequence Specific Oligonucleotide Probes (SSOP) is an amplification method to type genetic mutations and polymorphisms. The basis of this method is the HLA locus specific amplification by PCR and the subsequent probing of this product by SSOP. The sequence specific oligonucleotide probe is usually 15-20 bases in length and the probes will anneal to their complementary target sequences in the sample DNA. The SSOP approach consists of DNA extraction from peripheral blood and the subsequent amplification of 40–200ng of DNA with primers specific for nonpolymorphic regions surrounding the polymorphic locus to be tested followed by hybridization of the preamplificated DNA with locus-specific fluorescent-labeled probes [20]. As multiple related alleles exhibit few sequence differences, this assay generally requires a two-step approach with DNA resequencing of samples from patients who test positive after the first test. High-resolution *HLA* testing is needed to discriminate closely related alleles, such as HLA-B*57:02 and HLA-B*57:03, which are not associated with abacavir hypersensitivity. The expected time for this analysis is approximately 5h. Two kits are commercially available for SSOP with blood samples: HLA-B Dynal RELI™ SSO (Dynal Biotech Ltd, Invitrogen, UK) and LABtype^®^ SSO typing test (One Lambda Inc., Conoga Park, CA). The cost for reagents is approximately 35 Euros per reaction. 

### Sequence Based Typing (SBT)

The sequence based typing is a method characterized by specific amplification and subsequent resequencing. The SBT was one of the first molecular test able to discriminate the HLA-B*57:01 allele. Indeed, in 1999 a new high-resolution molecular typing method, termed SBT, was developed [21]. For this assay, genomic DNA was extracted from peripheral blood, and approximately 500–750ng of DNA are necessary for the analysis. The high-resolution typing of the *HLA-B* locus consists of a first amplification step that generates a product of approximately 1 kb corresponding to exon 2, intron 2, and exon 3. This PCR is followed by a second step of direct automated cycle sequencing. This approach permits high-resolution *HLA-B* subtypes of a DNA sample in 24h. Although this technique is more sensitive than serological tests, it is the most expensive typing method in terms of time, labor, and cost [22]. Indeed the SBT cannot be easily available in all laboratories [23].

### Sequence Specific Primers (SSP)

The method of sequence specific primers PCR (SSP-PCR) requires allele-specific primers that completely anneal with the DNA template. In typing by PCR amplification with sequence-specific primers (PCR-SSP), typing specificity is part of the amplification step, which makes the technique almost as fast as serological tissue typing. The amplicons will be separated by agarose gel electrophoresis.

Mallal and Colleagues [23] developed a quick test to detect HLA-B*57:01 using SSP in 2005. This protocol facilitates the rapid typing of HLA-B*57:01. Genomic DNA samples are extracted from blood samples, and 100 ng of genomic DNA are currently needed for the test. A multiplex PCR is performed to discriminate HLA-B*57:01 and the related B57 subtypes such as HLA-B*57:02, HLA-B*57:03, HLA-B*57:04, HLA-B*57:05, HLA-B*57:06 and HLA-B*57:09. The SSP cannot discriminates between HLA-B*57:01 and some low frequency *HLA-B* alleles. The PCR products are separated using agarose gel electrophoresis, and results are interpreted on the basis of positive or negative amplification of the HLA-B*57:01 locus as well as the HLA-B*57 locus. This also allows the discrimination of HLA-B*57:01 and the commonly occurring HLA-B*57:02 and HLA-B*57:03 alleles. As internal control for PCR, the SSP involves the use of two additional primers for *HGH *amplification.

A multicenter survey demonstrated the optimal specificity (100%) but imperfect sensitivity (99.3%) of this method [24]. Different kits are commercially available for blood samples: Micro SSP™ HLA DNA Typing Trays (One Lambda Inc., Conoga Park, CA), Olerup SSP® HLA typing kits (Qiagen, Vienna, Austria) and GENEQUALITY HLA-B*57:01 (AB ANALITICA srl, Padova – IT).

This method is quick and inexpensive (€15-30), representing a good choice to genotype patients prior to treatment with abacavir. Importantly, the assay take less than 3h to determine the genotype, and it can be performed also in small molecular laboratories, promoting the availability of the test in multiple countries. 

### SSP and Capillary Electrophoresis

The sequence specific primers PCR (SSP-PCR) can be also applied to capillary electrophoresis in order to increase sensibility and automation. This method requires a labelled allele-specific primers and the amplicons will be separated by direct capillary electrophoresis. The SSP-PCR and capillary electrophoresis is more sensitive the traditional SSP. In 2010, our group developed a new SSP-PCR assay based on fluorescence detection through capillary electrophoresis (CE) [25]. This technique has the important advantage to allow also the use of non-infective samples like saliva. Genomic DNA is extracted from buccal swabs, and less than 5 ng of DNA are needed for the analysis. The PCR is performed using the same primers for the standard agarose SSP-PCR method [23] with the forward primers labeled with 6-FAM and the PCR conditions optimized to increase the sensitivity of the reaction. CE allowed an easier interpretation of the data and makes possible the automation of the analytical protocol improving the throughput of the DNA analysis. The method shows a high sensitivity making possible the process of samples poorly concentrated (0,25 ng of DNA) such as DNA extracted by saliva and buccal swabs [22,23]. It is important to outline that the sensibility of this assay allows the use of very low amounts of DNA (0.25 ng) extracted from buccal swabs, which are much less infective. The main advantages of fluorescence typing methods include higher sensitivity and reproducibility and better automation of the analytical process, making genotyping of the HLA-B*57:01 allele rapid, economic, and easier. Moreover, the sensibility of this method can promote the use of noninfective sources of DNA, such as buccal swabs and saliva, increasing the availability and accessibility of the test with a reduction of costs for the shipment of samples from clinical centers to the laboratory. The average cost of this test is 50–70 Euros per sample. The test result can be obtained within 5 h of receipt of the sample.

### Real Time PCR

The Real Time PCR is a fast and direct method to amplify and analyze the genomic DNA. The HLA-B*57:01 allele can be identified by real-time PCR [26]. This protocol consists of a first step of PCR to amplify the entire DNA sequence and then a further analysis to determine the exact genotype using SYBR Green fluorescence through real-time PCR. Genomic DNA is extracted from peripheral blood samples, and 50 ng of DNA are used to performed the *HLA-B*-specific amplification between the first and third introns of the gene, which generates an amplicon of 922 bp. Preamplification of *HLA-B* results in an efficiency of 98%, whereas when genomic DNA was directly used as the template in Q-PCR, a significantly lower efficiency is observed. This assay can discriminate all twelve sequences deposited for HLA-B*57:01 (B*57:01:01-B*57:01:12), although alleles B*57:06, B*57:08, B*57:10, B*57:13-15, B*57:18-20, B*57:22-27, B*57:29-33, and B*57:35-37 cannot be excluded, as they are identical in the regions examined. It should be considered that these alleles are expected to be rare in the population [27]. Compared to AS-PCR methods described elsewhere [28,22], this new assay represents a significant improvement in terms of specificity, as alleles B*57:02, B*57:03, B*57:04, B*57:05, B*57:07, B*57:09, B*57:12, B*57:16, B*57:17, B*57:34, B*55:14, and B*58:14 can now be discriminated. 

Three kits are commercially available: HLA B*57:01 Real-TM (Sacace Biotechnologies Srl, Como, Italy), Duplicα Real Time Reagent Set HLA-B*57:01 (EuroClone s.p.a., Milano, Italy) and HLA-B*57:01 Screening Test (COBAS® AmpliPrep/COBAS® TaqMan® System, Roche Diagnostic, s.p.a., Monza, Italy) with cost of about 15 Euros. The method can be easy implemented by virology units and the sample can be used for both viral load determination and HLA-B*57:01 testing. 

### Allele Specific-PCR (AS-PCR)

The allele specific PCR is a selective amplification of specific allele. This technique is characterized by an accurate designing of the primers that must match/mismatch to the allele at the 3’-end of the primer. In 2007, Mallal and colleagues developed a different protocol for the detection of the HLA-B*57:01 allele based on AS-PCR through gel electrophoresis [28]. Genomic DNA is extracted from peripheral blood samples, and 80 ng of input DNA are needed. AS-PCR requires the use of three allele-specific primers to amplify exons 2 and 3 of the *HLA-B* locus (130bp) and two additional primers to amplify *HGH *as an internal control for PCR (439bp). 

An alternative AS-PCR molecular assay has been developed for high-resolution melting (HRM) through Real-Time PCR. HRM is a post-PCR method enabling genomic researchers to analyze genetic variations (single nucleotide polymorphisms (SNPs), mutations, methylations) in PCR amplicons. HRM characterizes nucleic acid samples based on their disassociation (melting) behavior. Samples can be discriminated according to their sequence, length, GC content. or strand complementarity. Even single base changes such as SNPs can be readily identified. First, PCR is performed to amplify the DNA region of interest. After the PCR process, the HRM analysis begins. The process is a precise warming of the amplicon DNA from approximately 50°C up to approximately 95°C. During this process, the melting temperature of the amplicon is reached, and the two strands of DNA separate or “melt.” It is possible to monitor this process in real time by using a fluorescent dye on a real-time thermocycler. Such fluorescent dyes bind specifically to double-stranded DNA, and at the beginning of the HRM analysis, there is a high level of fluorescence in the sample because of the billions of copies of the double-stranded amplicon. When the sample is heated and the two strands of the DNA melt, the presence of double-stranded DNA decreases, and thus, fluorescence is reduced. The presence of DNA variations, or in this case, the presence of DNA sequences compatible with HLA-B*57:01 can be detected through alterations of the normal temperature at which the DNA strands melt [28]. 

The method provides 100% sensitivity and specificity for the detection of HLA-B*57:01 and it is also applicable for the detection of HLA-B*57:06, HLA-B*57:08, HLA-B*57:10, HLA-B*55:14, and HLA-B*58:14. 

## CONCLUSIONS

In this review, we summarized the pharmacogenetic tests currently available for abacavir. The HLA-B*57:01 screening test minimizes potential abacavir-related toxicities by identifying patients who may be at risk of developing an HSR (Table **1**). With approximately 33 million people worldwide living with HIV/AIDS [29], minimizing the adverse effects of antiretroviral therapy is critical to controlling the infection and maintaining treatment adherence. HLA-B*57:01 testing to prevent abacavir hypersensitivity is cost-effective [30,31]. In one study, HLA-B*57:01 testing resulted in a cost-effectiveness ratio of $36,000 per quality-adjusted life expectancy compared to no testing [29]. Although these results represent a good example of translating research into clinical practice, additional efforts should be done to further improve the cost-effectiveness of genetic test as well as the access to the test. The cost-effectiveness of a pharmacogenomics strategy involves many different factors that need to be deeply considered. In this respect, in the case of abacavir, the development of many tests based on differently technology significantly improves the accessibility and availability of the test. Recent advances in molecular research provide several methods to detect HLA-B*57:01 allele (Tables **2** and **3**), offering different approaches meeting the specific needs of centers. The laboratories, different in terms of equipment, experience and automation, can choose the most fitting method on the basis of available technologies and/or preferred type of samples. This represents a driver for improving the cost-effectiveness of pharmacogenetic tests of abacavir as well as of other drugs. Furthermore, the use of noninfective sources of DNA can promote the centralization of testing laboratories by permitting different clinical centers to send samples to large and expert laboratories. Currently, it is possible to store DNA as well as buccal swabs or blood samples for many days, offering the possibility of not processing the samples immediately after collection. Strategies allowing a better automation of the tests should be encouraged to make possible the processing of a huge number of samples concurrently with a significant reduction of the costs of shipment and testing. However, the level of scientific stringency applied to the assay will depend on the knowledge accumulated about the PGBM as well as the implication of its use (context) [32]. Analytical performance criteria should be defined and justified in a pragmatic way so as to be proportionate to the stage of development and to risks and benefits of its intended use ("fit-for-purpose"). 

## Figures and Tables

**Table 1.** Frequency of HLA-B*57:01 allele in European Population

	White		Black		America Indian		Asian		Other		Overall	
Country	N° (positive)	HLA-B*57:01 prevalence % (SE)	N° (positive)	HLA-B*57:01 prevalence % (SE)	N° (positive)	HLA-B*57:01 prevalence % (SE)	N° (positive)	HLA-B*57:01 prevalence % (SE)	N° (positive)	HLA-B*57:01 prevalence % (SE)	N° (positive)	HLA-B*57:01 prevalence % (SE)
Italy	1545 (98)	6.34 (0.62)	72 (1)	1.39 (1.38)	26 (1)	3.85 (3.77)	5 (0)	0.00 (22.36)	18 (0)	0.00 (10.12)^b^	1666 (100)	6.00 (0.58)
Italy 2^a^	540 (29)	5.7 (0.97)	/	/	/	/	/	/	/	/	540 (29)	5.7 (0.97)
Switzerland	325 (33)	10.15 (1.68)	51 (0)	0.00 (3.57)^b^	4 (1)	25.00 (21.65)	13 (1)	7.69 (7.39)	5 (0)	0.00 (22.36)^b^	398 (35)	8.79 (1.42)
UK	618 (49)	7.93 (1.09)	770 (2)	0.26 (0.18)	26 (0)	0.00 (7.00) b	40 (2)	5.00 (3.45)	40 (3)	7.50 (4.17)	1494 (56)	3.75 (0.49)
Spain	1103 (70)	6.35 (0.73)	17 (0)	0.00 (10.71)^b^	68 (1)	1.47 (1.46)	1 (0)	0.00 (50.00)	0 (0)	1.91 (12.61)	1189 (71)	5.97 (0.69)
Portugal	108 (2)	1.85 (1.30)	40 (0)	0.00 (4.55)^b^	0 (0)	3.48 (4.98)	1 (0)	0.00 (50.00)	5 (0)	0.00 (22.36)^b^	154 (2)	1.30 (0.91)
France	1798 (122)	6.79 (0.59)	492 (2)	0.41 (0.29)	13 (0)	0.00 (13.87)^b^	39 (1)	2.56 (2.53) b	3 (0)	0.00 (28.87)^b^	2345 (125)	5.33 (0.46)
Germany	1717 (132)	7.69 (0.64)	94 (0)	0.00 (1.94)^b^	4 (1)	25.00 (21.65)	39 (2)	5.13 (3.53)	23 (0)	0.00 (7.92)^b^	1877 (135)	7.19 (0.60)
Ireland	142 (8)	5.63 (1.94)	18 (0)	0.00 (10.12)^b^	0 (0)	3.48 (4.98)	6 (0)	0.00 (20.41)	0 (0)	1.91 (12.61)	166 (8)	4.82 (1.66)
Finland	93 (2)	2.15 (1.50)	4 (0)	0.00 (25.00)^b^	0 (0)	3.48 (4.98) b	1 (0)	0.00 (50.00)^b^	2 (0)	0.00 (35.36)^b^	100 (2)	2.00 (1.40)
The Netherlands	229 (16)	6.99 (1.69)	29 (0)	0.00 (6.28)^b^	20 (1)	5.00 (4.87)	4 (0)	0.00 (25.00)^b^	12 (1)	8.33 (7.98)	294 (18)	6.12 (1.40)

SE: standard error.

a: Cases of our laboratory.

b: no HLA-B*57:01 positive patients in this category. SE was conservatively approximated.

Modified from [33].

**Table 2.** Genotyping HLA B*57:01 Allele Assays

Assay	Equipment required	Average reagent cost	Time of examination	References
Serological methods	Untouched Cells;Monoclonal antibodies	NA	NA	[14]
Flow cytometry	Cytoflow; Monoclonal antibodies (B17; 3E12)	>20€	2-3h	[18][19]
SSOP	Termocycler and strips for amplification/hybridization with sequence specific oligonucleotide probe; DNA sequencer (to confirm a positive samples)	>35€	5-8h	[20]
SBT	Termocycler for amplification with labelled primers/labelled sequence terminator; DNA sequencer	>30€	24h	[21][22]
SSP PCR	Termocycler for amplification with sequence specific primers;gel (2% agarose) electrophoresis	>30€	3h	[23][24]
SSP and Capillary Electrophoresis	Termocycler for amplification with labelled sequence specific primers; capillary electrophoresis	>50€	5h	[25]
Real Time PCR	Real-time PCR based on SYBR-Green fluorescence with allele-specific primers	>16€	2-3h	[26][27]
AS-PCR	Real-time PCR based on SYBR-Green fluorescence with allele-specific primers; HRM software	>30€	3-4h	[28]

**Table 3.** Commercially Available Kits

Kit	Methods	Characteristics	Company	References
HLA-B Dynal RELI™ SSO	SSOP	PCR and SSO hybridization on strips	Dynal Biotech Ltd, Invitrogen, UK	[34]
LABType® SSO Typing Tests	SSOP	PCR and SSO hybridization by Luminex platform	One Lambda Inc., Conoga Park, CA	[35]
Micro SSP™ HLA DNA Typing Trays	SSP	SSP PCR and agarose gel electrophoresis	One Lambda Inc., Conoga Park, CA	[36]
Olerup SSP® HLA typing kits	SSP	SSP PCR and agarose gel electrophoresis.The detection of four different groups of alleles of class I and II.	Qiagen, Vienna, Austria	[37]
GENEQUALITY HLA-B*57:01	SSP	SSP PCR through four steps of amplification. The first three steps detect the presence of HLA-B*57:01 allele, while the fourth step identifies one SNP in *HCP5* gene (internal control).	AB ANALITICA srl, Padova – IT	[38]
HLA-B*57:01 Real-TM	Real Time	Real-Time PCR characterized by one step of amplification.	Sacace Biotechnologies Srl, Como, Italy	[39]
DUPLICαReal Time Reagent SetHLA-B*5701	Real-time PCR based on SYBR-Green fluorescence	Amplification of HLA-B*57:01 allele and *HGH* gene as internal control typed by HRM	EuroClone s.p.a., Milano, Italy	[40]
HLA-B*5701 screening test COBAS® AmpliPrep / COBAS® TaqMan® System	Real Time	The platform combines the COBAS® AmpliPrep Instrument for automated sample preparation and the COBAS® TaqMan® Analyzer	Roche Diagnostic, s.p.a., Monza, Italy	[41]
